# The role of leptin in primary Sj**ö**gren syndrome: a clinical and histopathological assessment study

**DOI:** 10.3906/sag-2108-295

**Published:** 2021-12-11

**Authors:** Mustafa ERDOĞAN, Kayhan BAŞAK, Mehmet Engin TEZCAN

**Affiliations:** 1 Department of Rheumatology, Basaksehir Cam and Sakura City Hospital, Istanbul, Turkey; 2 Department of Rheumatology, Istanbul Kartal Dr. Lutfi Kirdar City Hospital, Istanbul, Turkey; 3 Department of Pathology, University of Health Sciences, Kartal Dr. Lutfi Kirdar City Hospital, Department of Pathology, Istanbul, Turkey

**To the editor**,

The underlying pathogenetic mechanisms of Sjögren syndrome (SjS) have not been elucidated yet [1]. Leptin is a glycosylated peptide structurally similar to some cytokines [2]. Leptin has proinflammatory effects via various mechanisms. It has been previously reported that serum leptin levels increase in some autoimmune diseases and that leptin levels are also associated with disease activity [3, 4]. In addition, leptin and its receptor in the salivary glands stimulate epithelial proliferation and modulate the local immune system with their autocrine effects [5]. In this study, we assessed the presence of leptin in minor salivary glands (MSG) of the patients with and without SjS. Additionally, we evaluated the association between leptin staining intensity and disease activity among patients with SjS.

We included patients who underwent MSG biopsy with the suspicion of SjS between 2013 and 2014. Among these patients, those who met the 2012 American College of Rheumatology Sjögren’s syndrome classification criteria (6) and had at least 50 mononuclear cell infiltrates in a 4 mm^2^ minor salivary gland section were classified as the SjS group. The rest of the patients who underwent MSG biopsy but did not meet the histopathological and/or clinical criteria for SjS were considered the control group.

Leptin staining was assessed by immunohistochemistry (Bio-Rad AbDSerotec, Oxford, UK) in the stroma, acinar and ductal epithelium, and staining intensities were graded by a semiquantitative method; no staining (score 0), mild (score 1), moderate (score 2), and diffuse (score 3). Definitions of leptin staining intensities were described as mild; focal staining of few cells in one focus, moderate; mild staining in more than one focus, diffuse; diffuse staining in all assessed sections (Figure 1). The sum of leptin staining in all areas was defined as total leptin staining. The SjS group was divided into 3 groups according to the focus scores (FS) (FS1: FS = 1, FS2: FS = 2, FS3: FS ≥ 3). In addition, patients’ disease activities were assessed by EULAR Sjogren’s syndrome disease activity index (ESSDAI) [7].

Visual (histograms, probability plots) and analytical methods (Kolmogorov-Smirnov test) were used to analyze the variables’ distribution and select the test method. Descriptive analyses were presented as mean or median according to their distribution patterns. A chi-square test was used for univariate analyses to identify variables associated with leptin staining and clinical features. Patients’ disease activities were assessed by ESSDAI [9]. The correlations between ESSDAI scores and leptin staining intensities were analyzed using Spearman’s test. A p value of <0.05 was considered statistically significant. No imputation was performed for missing values. Statistical analyses were done using the SPSS software v.20.

The study protocol was approved by the Local Ethics Committee of Dr. Lütfi Kırdar Training and Research Hospital – İstanbul.

The median age of the patients with SjS (n = 24) and the control group (n = 19) were 49.5 (24–78) and 53 (31–77), respectively. The mean ESSDAI score of the patients was 1.67 ± 1.44. The patients’ demographic and clinical characteristics and antibody test results are summarized in Table 1. The FSs of the patients were 1 in 25% (n = 6/24), 2 in 50% (n = 12/25) and ≥3 in 25% (n = 6/24). Leptin staining patterns of both groups were similar in acinar, ductal, or stromal areas (p = 0.827). However, among the patient group the proportion of patients with at least intermediate leptin staining intensity in stromal (in FS1 group: 33%, in FS2 group: 25%, in FS3 group: 100%), acinar (in FS1 group: 50%, in FS2 group: 33%, in FS3 group: 80%), and ductal (in FS1 group: 50%, in FS2 group: 66%, in FS3 group: 100%) areas were higher in the patients with FS3. There was a positive correlation between ESSDAI and leptin staining scores in the acinar area (p < 0.05; r = 0.406) regardless of focus scores. However, the correlations between disease activity and leptin staining in ductal areas (p: 0.1; r: 0.334), in stromal areas (p: 0.1; r: 0.184), and total leptin (p: 0.2; r: 0.268) staining was not significant.

We identified that majority of the patients in both groups had leptin staining in acinar (92% and 84%, respectively), ductal (94% and 84%, respectively), and stromal areas (96% and 100%, respectively) of their MSGs. Patients with higher FSs had significantly more intense leptin staining patterns. The possible explanations for the similar staining pattern in patients with an FS of ≤2 was the insufficient number of patients (β error) or absolute indifference. In contrast, a study by Erbasan F et al. argued that leptin has no role in primary SjS due to similar leptin staining patterns among patients with SjS and healthy controls. However, they did not compare patients according to the ESSDAI scores [8]. Thus, we still need more robust data to identify whether leptin has an inciting or prognostic role in pSS.

Limitations of our study were the lack of some clinical characteristics and metabolic parameters which may affect leptin metabolism and lack of leptin receptor staining patterns in MSG biopsies, which together with leptin staining could better define the role of leptin. Lack of power analysis was another limitation.

In conclusion, leptin staining properties were similar in both SjS and non-SjS groups. Additionally, the only clinical significance of leptin density in the MSG was in the acinar region.

## Figures and Tables

**Figure 1:**
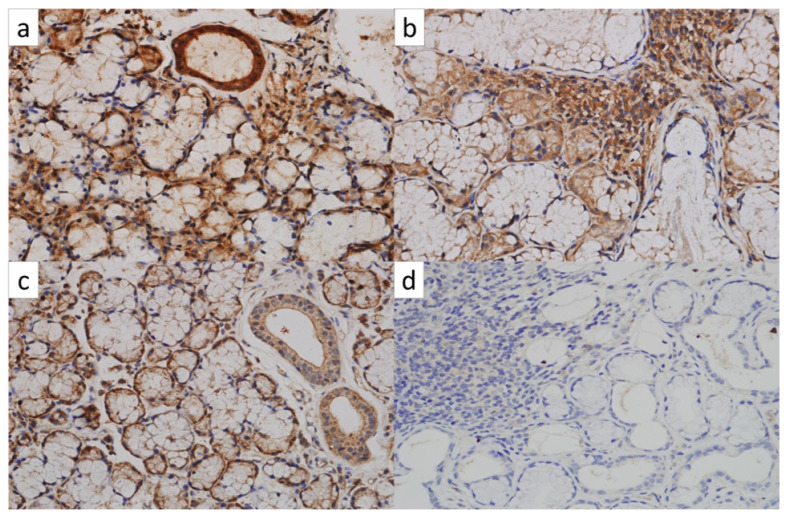
Examples for leptin staining patterns in minor salivary glands. a) Diffuse leptin staining (score 3) in acinar, ductal, and stromal areas in a minor salivary gland (× 400 magnification). b) Moderate (score 2) leptin staining in acinar and ductal areas in and diffuse leptin staining (score 3) in the stromal area in minor salivary gland (× 400 magnification). c) Diffuse leptin staining (score 3) in acinar and ductal minor areas and mild (score1) leptin staining in the stromal area in a salivary gland. d) No leptin staining in acinar and ductal areas and mild leptin staining (score 1) in the stromal area in minor salivary gland (× 200 magnification).

**Table 1:** Demographic, clinical features, and antibody results of patients.

	Patients with pSS (n = 24)	Patients without pSS (n = 19)	p value

**Median age (range)**	49.5 (24–78)	53 (31–77)	0,732

Sex **(F/M)**	24/0	17/2	0,189**

**Dry** mouth, n/N (%)*			0,455

No	8/24 (33)	9/19 (47)	

Yes	13/24 (54)	9/19 (47)	

**Dry eye, n/N (%)** *			0,14

No	6/24 (25)	9/19 (47)	

Yes	16/24 (67)	9/24 (47)	

**Arthralgia, n/N (%)** *			0.756**

No	11/24 (46)	10/19 (53)	

Yes	12/24 (50)	8/19 (42)	

**Arthritis, n/N (%)** *			0.679**

No	19/24 (79)	16/19 (84)	

Yes	4/24 (17)	2/19 (11)	

**Salivary gland swelling, n/N (%)** *			1 **

No	20/24 (83)	17/19 (90)	

Yes	2/24 (8)	1/19 (5)	

**Vasculitic skin rash, n/N (%)** *			1**

No	17/24 (71)	14/19 (74)	

Yes	5/24 (21)	4/19 (21)	

**Raynaud, n/N (%)** *			0,355**

No	18/24 (75)	17/19 (89)	

Yes	4/24 (17)	1/19 (5)	

**Fatigue, n/N (%)** *			0,476**

No	16/24 (67)	15/19 (79)	

Yes	6/24 (25)	3/19 (16)	

**Proteinuria, n/N (%)** *			1**

No	22/24 (92)	15/19 (79)	

Yes	1/24 (4)	1/19 (5)	

**Rheumatoid factor**			
Negative, n/N (%)	16/24 (67)	10/19 (53)	
**Positive, n/N (%)**	6/24 (25)	2/19 (11)	0,681**
**Unknown, n/N (%)**	2/24 (8)	7/19 (37)	
**Antinuclear antibody**			
Negative, n/N (%)	13/24 (54)	10/19 (53)	
**Positive, n/N (%)**	8/24 (33)	6/19 (32)	0,970
**Unknown, n/N (%)**	3/24 (13)	3/19 (16)	
**Anti SS-A**			
Negative, n/N (%)	10/24 (42)	12/19 (63)	
**Positive, n/N (%)**	3/24 (13)	4/19 (21)	1**
**Unknown, n/N (%)**	11/24 (46)	3/19 (16)	
**Anti SS-B**			
Negative, n/N (%)	12/24 (50)	12/19 (63)	
**Positive, n/N (%)**	1/24 (4)	1/19 (5)	1**
**Unknown, n/N (%)**	11/24 (46)	6/19 (32)	

* Absolute ratios and numbers of patients presented without correction for missing.

** Fisher’s Exact Test.
